# Enhanced photon emission from a double-layer target at moderate laser intensities

**DOI:** 10.1038/s41598-020-65778-4

**Published:** 2020-06-01

**Authors:** Martin Jirka, Ondrej Klimo, Yan-Jun Gu, Stefan Weber

**Affiliations:** 1 Institute of Physics of the CAS, ELI-Beamlines Project, Na Slovance 2, Prague, 182 21 Czech Republic; 2 Faculty of Nuclear Sciences and Physical Engineering, Czech Technical University in Prague, Brehova 7, Prague, 115 19 Czech Republic; 3 Institute of Plasma Physics of the CAS, Za Slovankou 1782/3, Prague, 182 00 Czech Republic; 4 School of Science, Xi’an Jiaotong University, Xi’an, 710049 China

**Keywords:** Laser-produced plasmas, Plasma-based accelerators

## Abstract

In this paper we study photon emission in the interaction of the laser beam with an under-dense target and the attached reflecting plasma mirror. Photons are emitted due to the inverse Compton scattering when accelerated electrons interact with a reflected part of the laser pulse. The enhancement of photon generation in this configuration lies in using the laser pulse with a steep rising edge. Such a laser pulse can be obtained by the preceding interaction of the incoming laser pulse with a thin solid-density foil. Using numerical simulations we study how such a laser pulse affects photon emission. As a result of employing a laser pulse with a steep rising edge, accelerated electrons can interact directly with the most intense part of the laser pulse that enhances photon emission. This approach increases the number of created photons and improves photon beam divergence.

## Introduction

Using today’s laser systems, electrons can be accelerated up to 8 GeV in 20 cm long capillary discharge waveguide^1^. When a bunch of accelerated electrons collides head-on with an intense laser pulse, these electrons will emit high-energy photons due to the inverse Compton scattering^2,3^. One of the goal of the scientific research nowadays is thus the realization of the all-optical compact source of high-energy *γ*-ray beam^4,5^. However, the efficiency of such a source depends on the properties of the electron and laser beams and on precise alignment of their interaction. Probability of photon emission is characterized by the parameter ${\chi }_{e}=\gamma /{E}_{\text{S}}\sqrt{{({\bf{E}}+{\bf{v}}\times {\bf{B}})}^{2}-{({\bf{v}}\cdot {\bf{E}}/c)}^{2}}$, where *γ* is the relativistic factor of the emitting particle (electron), ${\bf{E}}$ and ${\bf{B}}$ are the electric and magnetic fields, ${\bf{v}}$ is the particle velocity and $c$ is the speed of light in SI units^6^. In previous equation ${E}_{\text{S}}$ is the Sauter (Schwinger) limit field ${E}_{\text{S}}={m}_{e}^{2}{c}^{3}/(e\hslash )\approx 1.33\times {10}^{18}\,\text{V}/\text{m}$, *m*_*e*_ is the electron rest mass, $e$ is the elementary (positive) charge and $\hslash$ is the reduced Planck constant^7,8^. This parameter is maximized, when the electron is colliding head-on with the laser pulse. In such a case, the value of ${\chi }_{e}$ can be approximated as ${\chi }_{e}\approx 2\gamma {E}_{0}/{E}_{\text{S}}$, where ${E}_{0}$ is the amplitude of the laser field^9^. In such a case photon emission probability is only controlled by the energy of the incoming electron and the amplitude of the laser field.

Electrons in plasma can be accelerated by Laser Wake-Field Acceleration (LWFA) or Direct Laser Acceleration (DLA) mechanisms^10–12^. The latter becomes more important in the case of plasma densities higher than 10^20^ cm^−3^ and intensities going beyond today’s world record (>10^22^ W/cm^2^)^13–17^. To achieve such a high intensity, the laser pulse has to be tightly focused that will result in rapid diffraction of the laser field. Thus, the higher plasma density is required to compensate for diffraction in this case.

Nevertheless, head-on collision remains an issue from the experimental point of view due to the spatio-temporal alignment of the interaction^2^. This can be overcome by employing a plasma mirror. As the laser pulse impinges on the over-dense plasma mirror, it is reflected and thus previously accelerated electrons can interact with a counter-propagating laser field that leads to efficient photon emission^5,18^. This double-layer interaction setup can be further optimized by tuning the target properties (density, thickness) with respect to the laser intensity and focal spot radius to create the highest number of high-energy photons^19–27^.

**Figure 1:**
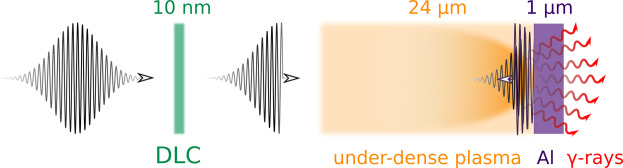
Interaction setup: the laser pulse gains a steep front edge after passing through the Diamond-Like-Carbon (DLC) layer (green). In the next stage, it accelerates electrons in the under-dense target and collides with them as it is reflected from Al plasma mirror. As a result of this interaction, *γ*-ray photons are emitted.

In this paper, we study photon emission in such an interaction scheme when various temporal profiles of the incoming laser pulse are assumed. For efficient photon production in a laser-electron collision it is crucial for the electron to get in the highest intensity region. As the electron enters the laser filed, it starts losing energy and thus can be expelled by the ponderomotive force before reaching the laser field amplitude. This effect that acts against efficient photon emission can be overcome by employing an appropriately tailored temporal profile of the laser pulse. The laser pulse with a steep front edge ensures that accelerated electrons will interact directly with the most intense part of the laser pulse and that consequently enhances photon emission, see Fig. 1. To our knowledge, the technique allowing direct shaping of the temporal profile of the femtosecond intense laser pulse while its frequency and intensity remain preserved has not yet been developed. Therefore, in the case of the current multi-petawatt laser systems, the laser pulse with a steep rising edge can only be realized by the preceding interaction with a dense and thin plasma foil^28^. Using numerical simulations we therefore present how the laser pulse that acquires a steep rising edge affects photon emission in the double-layer interaction setup.

## Results

To analyze photon emission in this interaction setup, we have performed 2D Particle-In-Cell (PIC) simulations in the code EPOCH^29,30^. At first, we considered the interaction of the laser pulse with 24 *μ*m-thick under-dense target containing electrons and protons of a density $0.1{n}_{\text{c}}$, where ${n}_{\text{c}}={\omega }_{0}^{2}{m}_{e}{\varepsilon }_{0}/{e}^{2}$ is the critical electron density and ${\varepsilon }_{0}$ is the vacuum permittivity. At the rear side of the under-dense target, 1 *μ*m -thick Al^11+^ foil of the electron density $385{n}_{\text{c}}$ is attached. The density is lower than the real density of solid aluminum due to computational constraints, nevertheless it does not have any significant influence on our results. This part of the double-layer target serves as a reflecting mirror for the laser pulse. The incoming laser pulse has a wavelength of 805 nm and Full-Width-At-Half-Maximum duration of $\tau =30\,\text{fs}$. The peak intensity ${I}_{0}=5\times {10}^{21}\,\text{W}/{\text{cm}}^{2}$ of the focused laser beam corresponds to the normalized laser amplitude ${a}_{0}=e{E}_{0}/({m}_{e}{\omega }_{0}c\mathrm{)}=45$ where ${\omega }_{0}$ is the laser angular frequency. These laser parameters are well within the capabilities of today’s laser systems such as J-Karen-P^17^.

**Figure 2:**
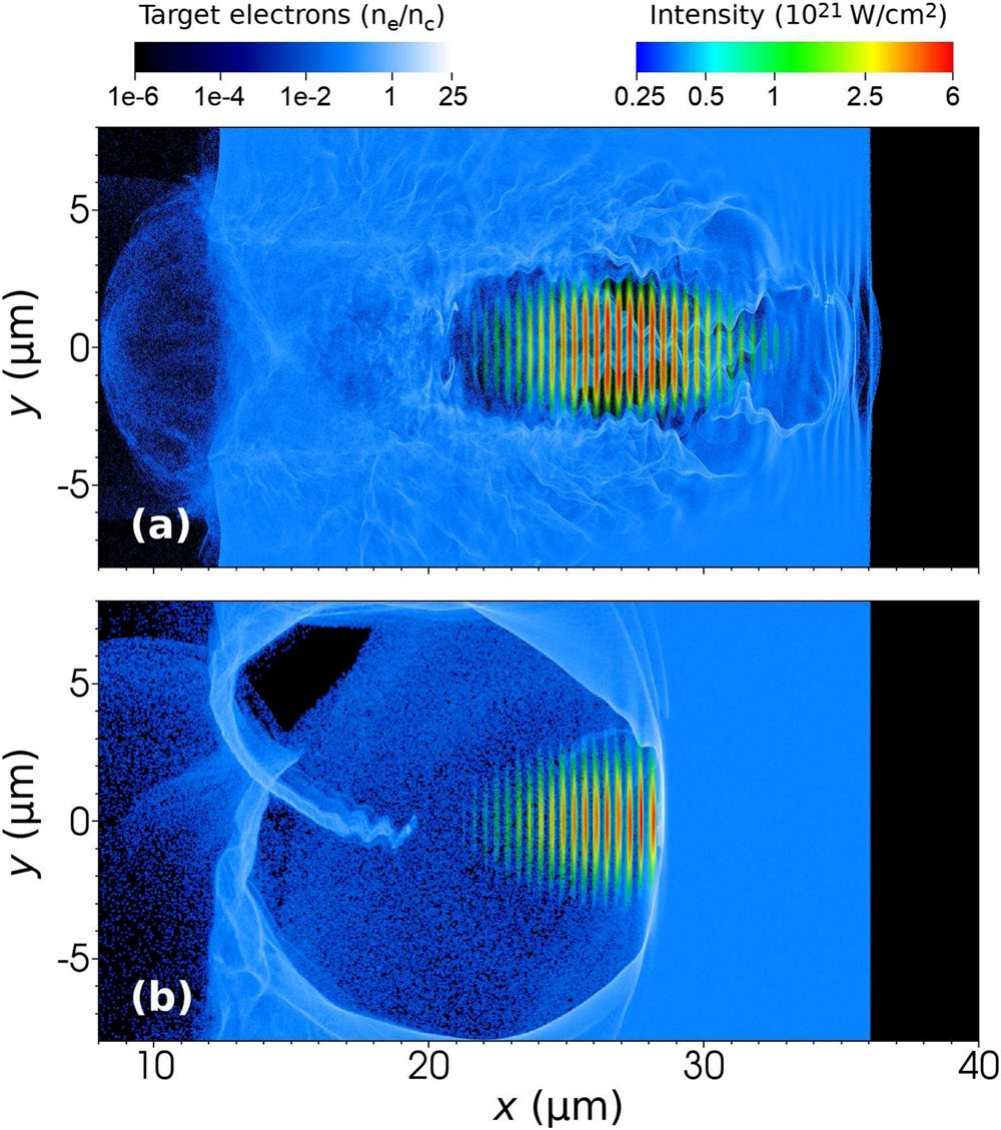
The intensity of the laser pulse and the density of target electrons in simulation Setups I and II in which the laser pulse has either (**a**) the Gaussian or (**b**) perfectly tailored temporal profile, respectively. The latter case was modelled by cutting the front part of the laser pulse.

At first, we have compared the interaction in which the laser pulse has either the Gaussian (Setup I) or perfectly tailored (Setup II) temporal profile of the laser pulse, as shown on snapshots from PIC simulations in Fig. 2a,b, respectively. The latter case was modelled by cutting the front edge of the laser pulse so that the electric field was equal to zero up to one-quarter of the laser period before the peak amplitude. Such a beam therefore delivers by almost 50% less energy onto the target compared to the previous case.

Setup I, i.e. when the laser pulse has the Gaussian temporal envelope, see Fig. 2a, represents the interaction of the intense laser pulse with under-dense plasma. Such an intense laser pulse can propagate through the plasma with minimal loses of its energy. As can be seen from Fig. 2a, for such a configuration, the bubble for LWFA scheme of electron acceleration is not efficiently developed. However, part of the target electrons is accelerated by the DLA scheme as they are trapped inside the laser pulse field structure.

By contrast, employing a tailored laser beam leads to considerable enhancement of electron acceleration. This is represented by Setup II, shown in Fig. 2b. In such a case, the laser beam has both a shorter duration and a steeper rise of the front edge. As the laser pulse enters the plasma, the electrons are immediately expelled sideways due to the strong ponderomotive force. Since the protons are not expelled so rapidly, they form a positively charged bubble behind the laser pulse. The created electrostatic field pulls the electrons back towards the laser axis. These electrons therefore exhibit betatron oscillations, as can be seen in Fig. 2b near $x=18\,\mu \text{m}$ and $y=-\,1\,\mu \text{m}$. As a result, the bubble behind the laser pulse can fully develop in Setup II compared to the previous case as the laser pulse propagates through under-dense plasma.

**Figure 3:**
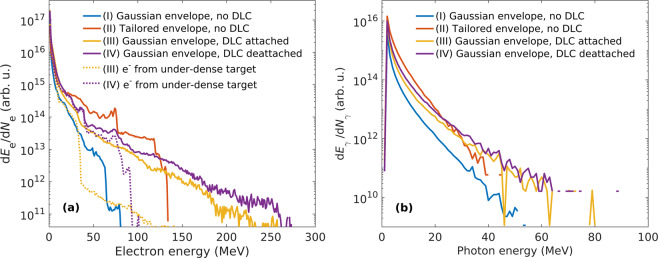
Energy distribution of (**a**) electrons at the time when the laser beam reaches the end of the under-dense target and of (**b**) photons at the end of the interaction. The laser beam has either (I) the Gaussian or (II) tailored temporal profile; or the laser beam with the Gaussian temporal profile is assumed while the DLC layer is (III) attached or (IV) detached from the under-dense target. Dotted lines represent only electrons from the under-dense target in corresponding runs.

The temporal profile of the laser pulse affects the motion of electrons in the plasma during the interaction and thus has an impact on their acceleration. We have seen in Fig. 2, that using the tailored laser beam profile enables more efficient acceleration of electrons via LWFA mechanism. This is confirmed in Fig. 3a showing electron energy spectra at the time when the laser pulse reaches the end of the under-dense target. Setups I and II are represented by lines I and II, respectively. From their comparison it is evident that much more electrons with higher energies are produced when the laser beam has a tailored temporal profile.

Photon emission can only be enhanced when these high-energy electrons collide with the laser field reflected from the aluminium foil attached at the end of the under-dense target, see Fig. 1. Therefore, the position of accelerated electrons with respect to the laser pulse as well as their energy are the key factors that affect photon emission. In Fig. 3b we present the energy spectra distribution of generated photons during the interaction. The cut-off energy of generated photons in Setup II is below 50 MeV even though electrons can be accelerated up to 130 MeV in this case, see Fig. 3a. However, the electrons having the highest energy are trapped in the front part of the tailored laser pulse, thus these DLA electrons can not collide with a sufficiently long part of the reflected laser pulse. High-energy photons are more likely generated by DLA electrons locked in the rear part of the laser pulse as well as by LWFA electrons dragged behind the laser pulse.

From the experimental point of view, the laser beam with a steep front edge can be realized by the interaction of the laser pulse with an ultra-thin solid-density foil, so-called plasma shutter, e.g. a Diamond-Like-Carbon (DLC) foil^31–36^. Since the foil is over-dense for the incoming laser pulse, the front part of the laser pulse is reflected. As the peak of the laser pulse impinges upon the foil surface, the relativistic mass of electrons suddenly increases causing the foil to become relativistically transparent for the rest of the laser pulse. Therefore, the laser pulse gains a steep front edge after passing through the foil.

**Figure 4:**
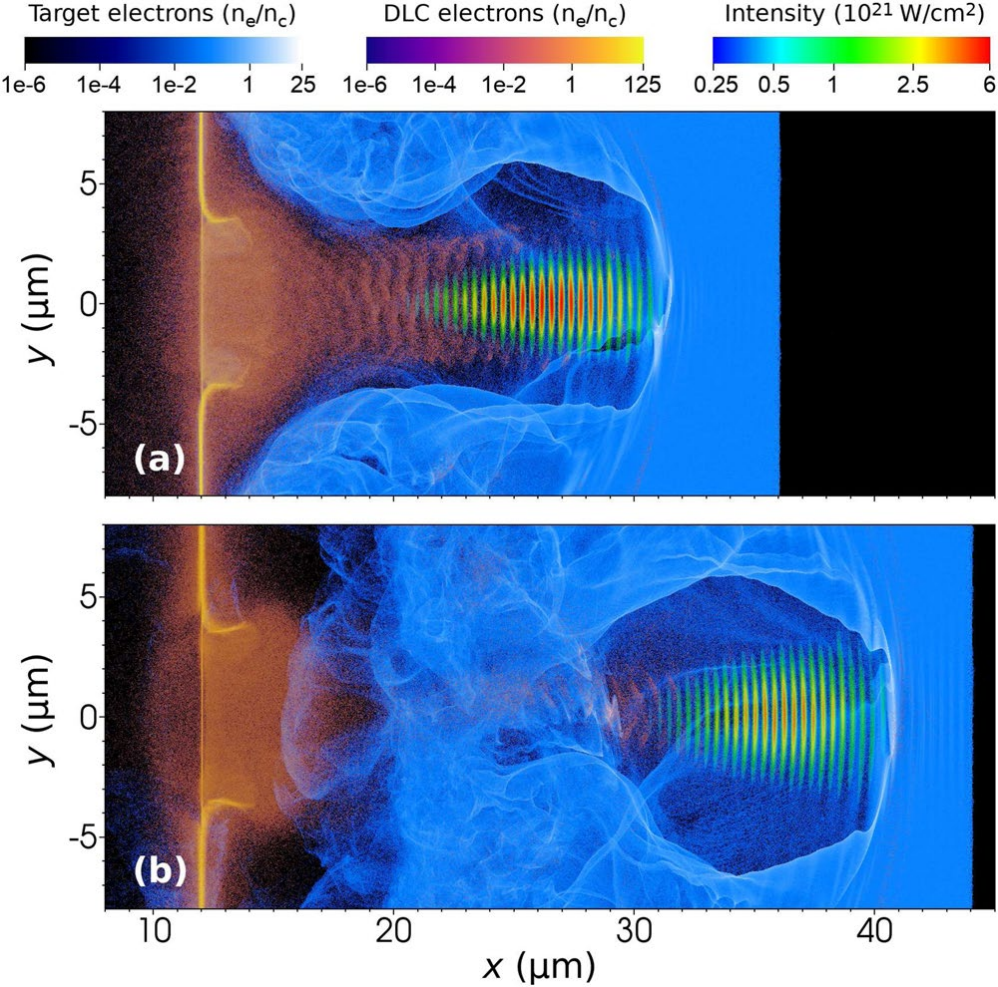
The density of DLC (orange) and target (blue) electrons and the laser intensity in simulation Setups III and IV in which the DLC layer is either (**a**) attached or (**b**) detached from the under-dense target, respectively. The incoming laser pulse has the Gaussian temporal profile.

**Figure 5:**
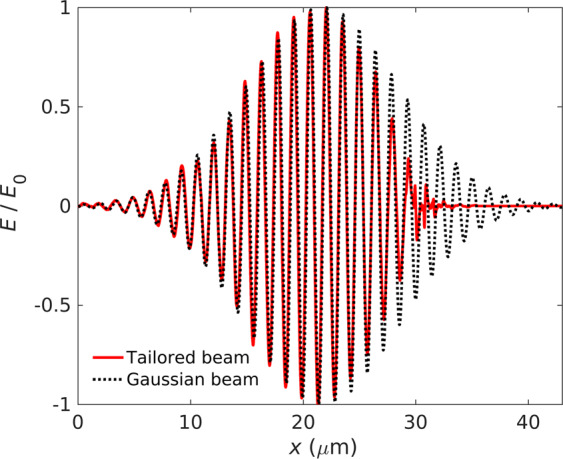
The result from 2D PIC simulation: *E*_*y*_ component of the laser pulse before (black) and after (red) passing through the DLC layer.

In the following text, we present the results of electron acceleration and photon emission in PIC simulations in which the preceding interaction of the laser pulse with the foil is taken into account. At first, we have performed simulation for Setup III, in which a 10 nm-thin DLC foil is attached at the front side of the target^35,36^. The DLC electrons are depicted by orange color in Fig. 4a. The fully ionized DLC foil has the electron density $384{n}_{\text{c}}$. As the laser pulse initially having the Gaussian temporal profile passes through the DLC foil, it gets a steep front edge, as shown in Fig. 5. By cutting the front part of the laser pulse, it loses about 15% of its initial energy.

After passing the foil, the tailored laser pulse interacts with the double-layer target. The dynamics of DLC electrons negatively affects the acceleration of electrons originating in under-dense plasma. Electrons from under-dense plasma (blue) are immediately expelled by the laser pulse while the DLC ones (orange) are attracted by protons to compensate for the charge separation field created behind the laser pulse. For this reason, the electrons which are expelled sideways by the ponderomotive force can not form the bubble and thus are not trapped at the back of this structure, see Fig. 4a. Thus, the acceleration of electrons in under-dense plasma is efficiently reduced. It agrees with the electron spectrum represented by line III in Fig. 3a. It shows, that electrons belonging to the under-dense target (dotted line) have much lower cut-off energy than the DLC ones. In this configuration, the cut-off energy for photons is 80 MeV. As shown in Fig. 3a, there are much more DLC electrons with energy higher than 50 MeV. Therefore, mainly the DLC electrons located in the rear part of the laser pulse are the ones responsible for generation of high-energy photons.

As the main disadvantage of the previous interaction setup is that the laser wake-field structure can not fully develop in the under-dense target we propose another configuration, Setup IV, in which the DLC layer is initially detached from the double-layer target by a $8\,\mu \text{m}$ vacuum gap. This is illustrated in Fig. 4b. Due to the sufficiently large vacuum gap between the DLC layer (orange) and the under-dense target (blue), the DLC electrons do not have enough energy to overcome the potential induced at the surface of the foil and to enter the under-dense target and thus are not attracted by the protons. As a result, the DLC electrons do not prevent development of the bubble in plasma. Therefore, detaching the DLC layer from the under-dense target leads to a more efficient LWFA of electrons originating their motion in under-dense plasma. These electrons are trapped behind the laser pulse and thus they have the favourable position for emitting photons when they interact with the reflected laser pulse. As a result, more photons are emitted when the DLC layer is detached from the double-layer target, see Fig. 3b where lines III and IV represent the corresponding setups. However, the cut-off in the photon energy spectrum for Setup IV is still about 80 MeV despite the improvement in the electron energy spectrum cut-off. This is due to the fact that the most energetic electrons are the DLC ones locked in the front part of the laser pulse which do not have a chance to significantly contribute to photon emission. Nevertheless, employing the detached DLC layer allows creating the highest number of high-energy photons in comparison with all the Setups presented above, see Fig. 3b.

Even though employing the detached DLC layer causes faster diffraction of the laser pulse, compare the laser field structure in Figs. 2a and 4b, it does not considerably affect photon emission. The efficiency of photon emission depends on the electron energy and the experienced laser intensity. Since Setup IV employing the DLC foil can provide electrons that are accelerated to higher energies compared to Setup I and these can experience the higher laser intensity due to the steep rising edge of the laser pulse, the photon emission is more pronounced in this case.

**Figure 6:**
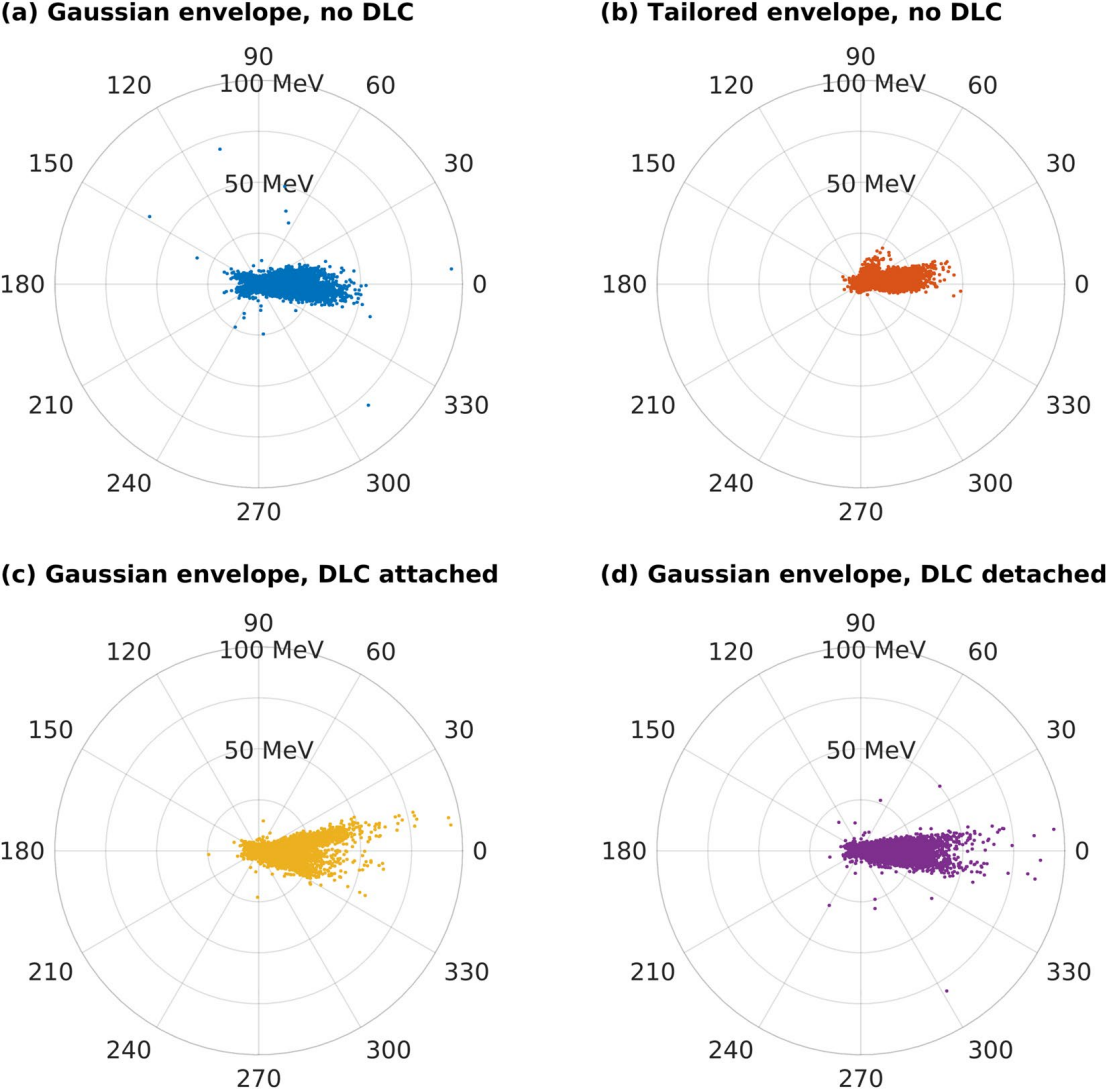
The angular energy distribution of photons for Setups I–IV at the end of the simulation. The laser beam has either (**a**) the Gaussian or (**b**) tailored temporal profile; or the laser beam with the Gaussian temporal profile is assumed while the DLC layer is (**c**) attached or (**d**) detached from the under-dense target.

Moreover, the angular characteristics of the emitted photon beam are also improved in Setup IV, see Fig. 6. As described above, when the DLC layer is employed and detached from the target (Setup IV), the LWFA mechanism can develop and more electrons are accelerated via this mechanism. Since the bunch of LWFA electrons is collimated, such a configuration results in a narrower angular distribution of emitted photons compared to Setup III, see Fig. 6(c,d) and Table 1.

**Table 1:** The number of photons ${N}_{\gamma }$, their mean energy $\langle {E}_{\gamma }\rangle$, conversion efficiency ${\eta }_{\gamma }$ of laser energy to photons and the photon beam divergence angle $\theta$ relative to the laser propagation direction for simulation Setups I–IV. Energy is normalized to the energy of the Gaussian laser pulse.

	I	II	III	IV
${N}_{\gamma }\mathrm{\ (1/}\text{m})$	$5.2\times {10}^{15}$	$2.5\times {10}^{16}$	$1.2\times {10}^{16}$	$1.5\times {10}^{16}$
$\langle {E}_{\gamma }\rangle (\text{MeV})$	1.52	2.12	1.66	1.89
${\eta }_{\gamma }\mathrm{\ ( \% )}$	0.035	0.23	0.088	0.12
$\theta \mathrm{\ (}\text{rad})$	0.32	0.28	0.35	0.26

The results for all presented Setups are summarized in Tab. 1: efficiency of photon emission, i.e. number of photons, their mean energy, efficiency of laser energy conversion to photons and the divergence of a photon beam with respect to the laser propagation direction. The number of photons is obtained from photon spectra taking into account photons with energy $\ge 0.5\,\text{MeV}$. Divergence of the photon beam is characterized by angle $\theta =\sqrt{\frac{1}{N}{\sum }_{i=1}^{N}\varDelta {\theta }_{i}^{2}}$ where $\varDelta {\theta }_{i}=\text{arctan}\frac{|{p}_{y,i}|}{{p}_{x,i}}$ and ${p}_{x,i}\ge 0$, ${p}_{y,i}$ are the components of photon momentum.

Employing the laser pulse with a steep front edge (Setup II) results in generating 5x more photons than for a laser pulse with the Gaussian temporal envelope (Setup I). The mean photon energy in such a case is about 40% higher and conversion efficiency is increased by a factor of six.

In Setup III, the number and mean energy of created photons are much lower than in Setup II as the presented DLC foil is not dense enough to create ideally tailored laser beam. However, the number and mean energy of created photons are still higher compared to Setup I.

For Setup IV the theory predicts photons with typical energy around 2 MeV^37^. This agrees with our results from PIC simulations. The conversion of the laser energy to photons is increased by a factor of 3.4 compared to Setup I. Optimizing the distance between the DLC layer and the target with respect to the target density can further enhance conversion efficiency. The length of a vacuum gap allows the DLC electrons to expand and thus reduce their number which enters the under-dense target. The optimal length of the vacuum gap is given by the parameters of the laser pulse (intensity, focal spot radius, temporal duration) and of the foil (density, thickness). If the gap is too small, DLC electrons can enter the under-dense target and prevent the formation of the wake-field structure. On the other hand, when the vacuum gap is extremely wide, the laser pulse may considerably diffract and thus the acceleration of target electrons becomes less efficient. For example, by performing a set of PIC simulations, we have found that the optimal length of the vacuum gap is about 4 *μ*m for the above-mentioned parameters. The conversion efficiency of laser energy to photons in such a case is four times higher compared to Setup I.

## Discussion

Up to this point, we have assumed the fixed target density while the distance from the DLC layer was varied. Increasing the plasma density leads to a creation of a higher number of photons by electrons from the under-dense target and thus more efficient laser energy conversion provided that the relativistic critical density *γn*_*c*_ is not reached. However, if the plasma density is too high then the laser pulse can be rapidly depleted. On the other hand, if an intense laser pulse propagates in near-critical-density plasma for a sufficiently long distance, it may undergo the effect of relativistic self-focusing that increases the laser intensity and reduces diffraction^38,39^. That, in turn, can lead to emission of photons with higher energy. The optimal propagation distance with respect to a given plasma density is therefore limited by these two effects^23,40,41^.

To assess the role of a higher plasma density, we have performed additional simulations, in which the target density has been increased by a factor of 10 from 0.1*n*_*c*_ to 1*n*_*c*_. Targets of such a density have been already demonstrated, e.g. refs. ^42,43^. Due to self-focusing, the laser pulse gains a smaller transverse profile and a higher peak intensity. In our case, the peak intensity of the laser field in 1*n*_*c*_ target is by 25% higher than in 0.1*n*_*c*_ one. Moreover, the DLA is more efficient at such a plasma density as it enables to accelerate a higher number of electrons. As a result, the laser energy conversion to photons is higher by a factor of 15 compared to Setup I, i.e. when the DLC layer is not considered. Employing the DLC layer is still feasible for such a dense target as it enhances photon production by a factor of 1.3. This confirms the applicability of our setup even for near-critical-density plasma targets.

The efficiency of laser energy conversion to photons in Setup IV is approximately the same as in the case when 20 pC LWFA electron bunch having energy 0.5 GeV collides with the laser pulse of the same properties as described above. In such a case we obtain ${\eta }_{\gamma }\approx \mathrm{0.10 \% }$ according to ref. ^44^ Although it is possible to achieve higher electron energies using LWFA compared to our setup, it might be complicated to reflect and focus the driving laser pulse to initiate photon emission^45,46^. The presented setup is therefore more robust as it encompasses both the acceleration and photon-emission stages while the latter does not rely on a focusing mirror.

It has been shown that photon emission in the interaction of a laser pulse with the under-dense target and reflecting plasma mirror can be enhanced by employing a laser pulse with a steep front edge. Such a beam can be created by the preceding interaction of the laser pulse with a thin solid-density foil, plasma shutter. The shaped laser pulse then propagates through the vacuum into the under-dense target in which electrons are accelerated via LWFA and DLA mechanisms. The vacuum gap between the foil and the target ensures that electrons dragged from this foil will not counteract the acceleration of electrons in the under-dense target. The accelerated electrons then interact with the most intense part of the laser pulse reflected from the plasma mirror. Therefore, employing the solid-density foil will result in a more efficient conversion of the laser energy to photons. For the parameters described above we obtained three times higher conversion efficiency and a narrower angular distribution of emitted photons compared to the interaction without the thin solid-density foil. This can be further improved by adjusting the density and thickness of the foil to provide the optimal temporal profile of the laser pulse. As the laser pulse loses its energy in the under-dense target very slowly, the length of the electron acceleration stage could be optimized with respect to the laser intensity to get the highest number of accelerated electrons.

## Methods

### Numerical modelling

To analyze the presented laser-plasma interaction we used the PIC code EPOCH in which photon emission is considered as a step-like quantum process^30^. For details about the implementation of photon emission into this code the reader is referred to ref. ^29^.

In 2D simulations of Setups I–IV, the box was spanning from 0 to 50 *μ*m in the *x*-direction and from −15 *μ*m to 15 *μ*m in the *y*-direction. Such a simulation domain was resolved with 22,320 × 13,392 cells. This is sufficient as for a density of 385*n*_*c*_ the plasma skin depth is about 6.5 nm. The spatial resolution remained unchanged for all other performed 2D simulations (e.g. parameter scan for the optimal length of a vacuum gap), while the size of the simulation box was enlarged. The laser pulse enters the box at a boundary $x=0\,\mu \text{m}$. The DLC layer of thickness 10 nm was located at $x=11.99\,\mu \text{m}$ (Setups III and IV) while the under-dense target was spanning from $12\,\mu \text{m}$ to $36\,\mu \text{m}$ (Setups I–III) or from $20\,\mu \text{m}$ to $44\,\mu \text{m}$ (Setup IV). At the rear side of the under-dense target a 1 *μ*m-thick Al^11+^ foil was attached. The laser pulse having the Gaussian temporal envelope propagates in the positive *x*-direction while being polarized along the *y*-axis. It is focused to a focal spot of radius ${w}_{0}=1.5\,\mu \text{m}$ located at $x=12\,\mu \text{m}$ in the simulation box.

## Data Availability

The datasets generated and analyzed during the current study are available from the corresponding author on reasonable request.
